# Multitarget mechanisms and clinical integration of traditional Chinese medicine in cardiac rehabilitation: a narrative review

**DOI:** 10.3389/fcvm.2026.1895271

**Published:** 2026-08-19

**Authors:** Yali Zhang, Jiahui Zhang, Shiwan Deng, Luting Zhao, Xiaoxia Yu

**Affiliations:** 1 Department of Rehabilitation and Pain Medicine, West China Hospital of Sichuan University—Ziyang Hospital (Ziyang Central Hospital), Ziyang, Sichuan, China; 2 Department of Respiratory and Critical Care Medicine, West China Hospital of Sichuan University—Ziyang Hospital (Ziyang Central Hospital), Ziyang, Sichuan, China; 3 Department of Rehabilitation Medicine, Sichuan University West China Hospital Tibet People’s Government in Chengdu Office Branch, Chengdu, Sichuan, China

**Keywords:** cardiac rehabilitation, Chinese herbal medicine, integration, traditional Chinese exercises, traditional Chinese medicine

## Abstract

Cardiovascular diseases (CVD) are a major global public health burden. Annually in the United States, over one million patients undergo arduous cardiac rehabilitation (CR) after experiencing cardiovascular events such as myocardial infarction (MI), percutaneous coronary intervention (PCI), coronary artery bypass grafting, cardiac valve surgery, and heart transplantation. Although CR can reduce mortality, it fails to fully address residual risks, including chronic inflammation, autonomic dysfunction, and psychological disorders. Meanwhile, low patient participation and completion rates remain a major clinical challenge. Traditional Chinese medicine (TCM) shows multi-target cardioprotective effects that can effectively complement CR. This review focuses on the theoretical compatibility, multi-target mechanisms, and clinical application of TCM integrated with CR. Clinically, TCM effectively compensates for the limitations of conventional CR. Traditional Chinese exercise (TCE) acts as an optimal bridging therapy to facilitate safe rehabilitation initiation in deconditioned populations prior to standard aerobic training. The application of Chinese herbal medicine (CHM) in complex metabolic and psychological comorbidities refractory to conventional pharmacotherapy yields prominent advantages in CR. During inpatient and early outpatient rehabilitation, acupuncture and acupoint stimulation modulate autonomic function, relieving postoperative angina and myocardial ischemia without increasing cardiac burden. Despite promising prospects, inconsistent syndrome classification standards and inadequate high-grade evidence remain obstacles to its global clinical adoption. This review therefore elaborates on the theoretical rationale and clinical evidence to promote the integration of TCM-CR as a novel holistic regimen for CVD rehabilitation.

## Graphical Abstract

**Figure:**
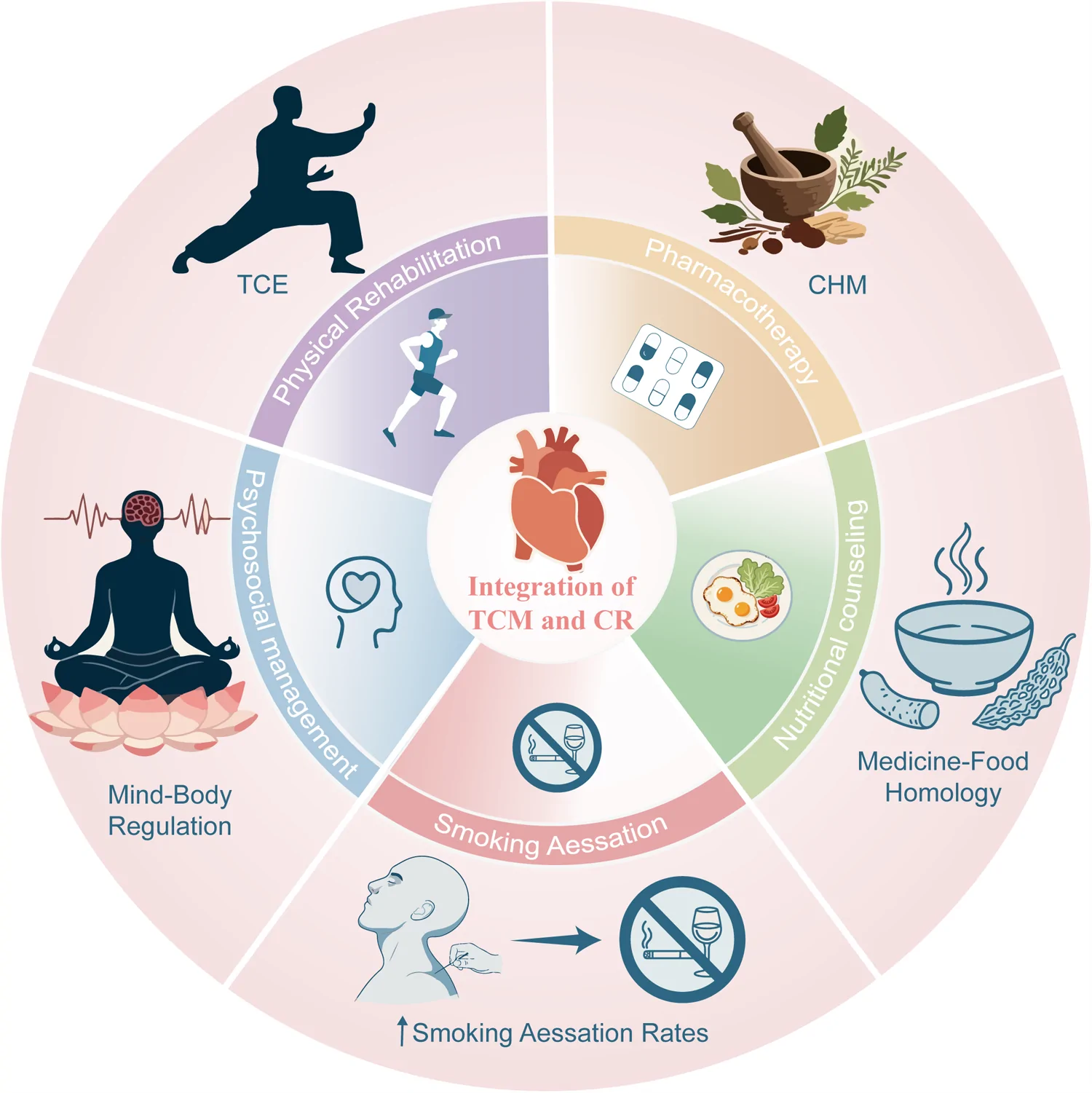
Integration of TCM and CR. CHM, Chinese herbal medicine; CR, cardiac rehabilitation; TCE, traditional Chinese exercise; TCM, traditional Chinese medicine.

## 1 Introduction

Driven by high prevalence and associated mortality, CVD have emerged as a critical global public health challenge. In 2023, the global prevalence of CVD reached approximately 626 million, resulting in 19.2 million related deaths (1). Annually in the United States, over one million patients undergo arduous CR after experiencing cardiovascular events such as MI, PCI, coronary artery bypass grafting, cardiac valve surgery, and heart transplantation. CR is a multidisciplinary, systematic and individualized intervention model that aims to provide secondary preventive strategies for patients with CVD, reducing all-cause mortality by 20% to 24% and significantly declining readmission rates (2, 3). However, global CR utilization and completion rates remain inadequate (4). Only 24% of eligible patients started CR within 21 days post-qualifying event, with merely 27% completing the full course of rehabilitation. Furthermore, conventional exercise-based CR provides insufficient reduction of residual risks. These primarily include chronic systemic inflammation, autonomic dysfunction, and psychological distress (3).

Therefore, there is an urgent need for strategies to address residual biological risks and improve utilization and completion rates. Over the past decade, evidence has indicated that the integration of TCM and CR has yielded more comprehensive therapeutic benefits. TCM exerts comprehensive and multi-targeted protective effects on CVD (5). However, existing studies predominantly focus on single interventions. There remains a scarcity of evidence regarding the combined application of TCE, CHM, and external TCM interventions in the treatment of CVD. Furthermore, the theoretical compatibility and potential mechanisms have not been fully elucidated, and significant gaps remain in clinical integration. This review evaluates the theoretical integration of TCM and CR, summarizes recent clinical advances, and analyzes current challenges and future perspectives, thereby providing actionable insights to advance this integration.

## 2 Theoretical compatibility between TCM and CR

Conventional CR is based on the biopsychosocial model, containing exercise, pharmacy, nutrition counseling, psychology, and risk factor modification (6). Rooted in a profound theoretical framework, TCM emphasizes holism and individualized treatment. It is highly consistent with the holistic intervention concept emphasized in conventional CR.

### 2.1 TCE and exercise

Conventional CR formulates individualized exercise prescriptions for patients based on precise assessments such as cardiopulmonary exercise testing and the 6-minute walk test. However, improving adherence to these prescriptions remains a significant challenge (7). Meanwhile, home-based CR yields superior adherence compared with center-based CR (3). As low-to-moderate intensity and whole-body regimens, TCE such as Tai chi and Baduanjin offer a compelling solution. In addition to improving physical function, TCE also plays a significant role in psychological regulation. Requiring no equipment, TCE can be easily performed at home, thereby providing a safe and convenient access for deconditioned patients, particularly the elderly or individuals with cardiac dysfunction. In conclusion, TCE elevates participation and completion rates of CR (8), representing a valuable complement to conventional exercise prescriptions in CR.

### 2.2 CHM and pharmacy

CHM is uniquely guided by syndrome differentiation, ensuring tailored prescriptions for individual patients. Research indicated that CHM possessed unique advantages in improving myocardial perfusion, regulating heart rhythm, and alleviating symptoms, thereby complementing conventional pharmacotherapy in CR (9). For example, formulations such as Compound Danshen Dripping Pills have shown promise in improving endothelial function (10). Meanwhile, formulated herbal products like Yangxinshi tablets have demonstrated efficacy not only in enhancing functional capacity but also in ameliorating CR-related psychological distress (11). Furthermore, certain progress has been achieved in the application of single Chinese herb extracts in CR (12–14). Collectively, these CHM provide a multifaceted pharmacological adjunct to standard CR protocols.

### 2.3 Medicine-food homology and nutrition counseling

Conventional nutritional guidelines for CR tend to overemphasize quantifiable biomarkers, including blood pressure, lipids, and fasting glucose, thereby overlooking patient heterogeneity. Traditional Chinese dietary therapy complements this by applying the theory of medicine-food homology. This approach provides an individualized nutrition strategy tailored to a patient's syndrome differentiation. For instance, foods traditionally used to combat fatigue (e.g., *yam*) provide prebiotics that improve energy metabolism in deconditioned patients. Conversely, foods used to manage hyperglycemia (e.g., *bitter melon*) offer mild hypoglycemic and sedative effects for those with metabolic syndrome and sympathetic hyperactivation (4). Notably, this dietary framework aligns closely with the 2024 American Heart Association scientific statement, which highlights personalized nutrition as an essential component of CR (6). Additionally, a systematic review of 99 studies revealed the anti-inflammatory and microbiota-modulating effects of TCM dietary patterns, which are characterized by high intake of whole grains and vegetables and low meat consumption. These effects translate into significant reductions in CVD risk and improved metabolic profiles (15). Ultimately, synthesizing TCM dietary strategies with established protocols like the Dietary Approaches to Stop Hypertension diet enhances individualized rehabilitation and drives long-term adherence.

### 2.4 Risk factor modification: smoking cessation and alcohol restriction

Smoking cessation and alcohol moderation are foundational to conventional CR, which have proven to significantly improve endothelial function and lower major adverse cardiovascular events (MACEs) (6). However, conventional smoking cessation methods are associated with low success and high relapse rates. Studies have indicated that although nearly half of U.S. adult smokers attempt to quit annually, fewer than 10% maintain abstinence for six months or longer (16).

Smoking and excessive alcohol consumption can induce systemic microinflammation, oxidative stress, and metabolic disturbances (17, 18). In TCM theory, these pathological manifestations essentially equate to the depletion of healthy qi and the intermingling of phlegm and blood stasis, which directly exacerbate the progression of CVD (19). To counteract these behaviors, TCM employs a multimodal strategy that extends beyond conventional counseling. For example, acupuncture and acupoint stimulation have shown promise in boosting sustained smoking abstinence rates by regulating neurochemical pathways underlying addiction (20). Importantly, by integrating these neuromodulation techniques with mind-body regulation, TCM simultaneously addresses physiological dependence and psychological cravings (21). This comprehensive, non-pharmacological supportive strategy not only alleviates withdrawal symptoms but also significantly enhances long-term adherence to behavioral modifications, thereby maximizing the overall efficacy of lifestyle intervention in CR.

### 2.5 Mind-body regulation for psycho-cardiology

Psychological distress, notably anxiety and depressive symptoms, is highly prevalent in patients with CVD and exacerbates disease progression via the neuroendocrine-immune regulatory network (22). Epidemiological studies show that over 40% of individuals with CVD present comorbid anxiety, while more than 30% develop depressive disorders; both independently predict adverse cardiovascular events (23). Standard CR has gradually incorporated the paradigm of psychocardiology, given the well-documented impact of psychological wellbeing on cardiovascular clinical outcomes. Despite the proven efficacy of mainstream psychological interventions such as cognitive behavioral therapy, their routine clinical use remains hampered by insufficient access and low patient participation.

The TCM theory of diseases induced by emotional disturbances aligns well with modern psychocardiology, underscoring that ideal CR demands combined physical and psychological interventions. Grounded in the core tenet of mind-body holism, TCM provides a distinctive theoretical framework for psychological regulation. Instead of only correcting cognitive biases, TCM modulates psychological status via somatic regulatory pathways. For instance, Tai Chi simultaneously suppresses the hypothalamic-pituitary-adrenal axis and improves parasympathetic tone, thereby attenuating physiological stress responses as well as psychological anxiety. Furthermore, TCM integrates emotional regulation therapies (e.g., cognitive redirection and Five Elements-based music therapy) to directly relieve emotional cravings and psychological distress (24). Moreover, acupuncture targeting specific neuromodulatory acupoints (GV20, EX-HN3, for example) can modulate neurotransmitter turnover, normalize sleep architecture, and mitigate mood disturbances (25). Furthermore, TCE including Tai Chi and Baduanjin integrate physical activity, breath regulation, and mental focus to induce the physiological relaxation response, thereby diminishing systemic sympathetic activation (26). This holistic mind-body intervention serves as an easily accessible, drug-free modality capable of disrupting the self-perpetuating cycle of psychological stress and worsening cardiac function.

## 3 Core mechanisms of TCM in CR

**Figure 1:**
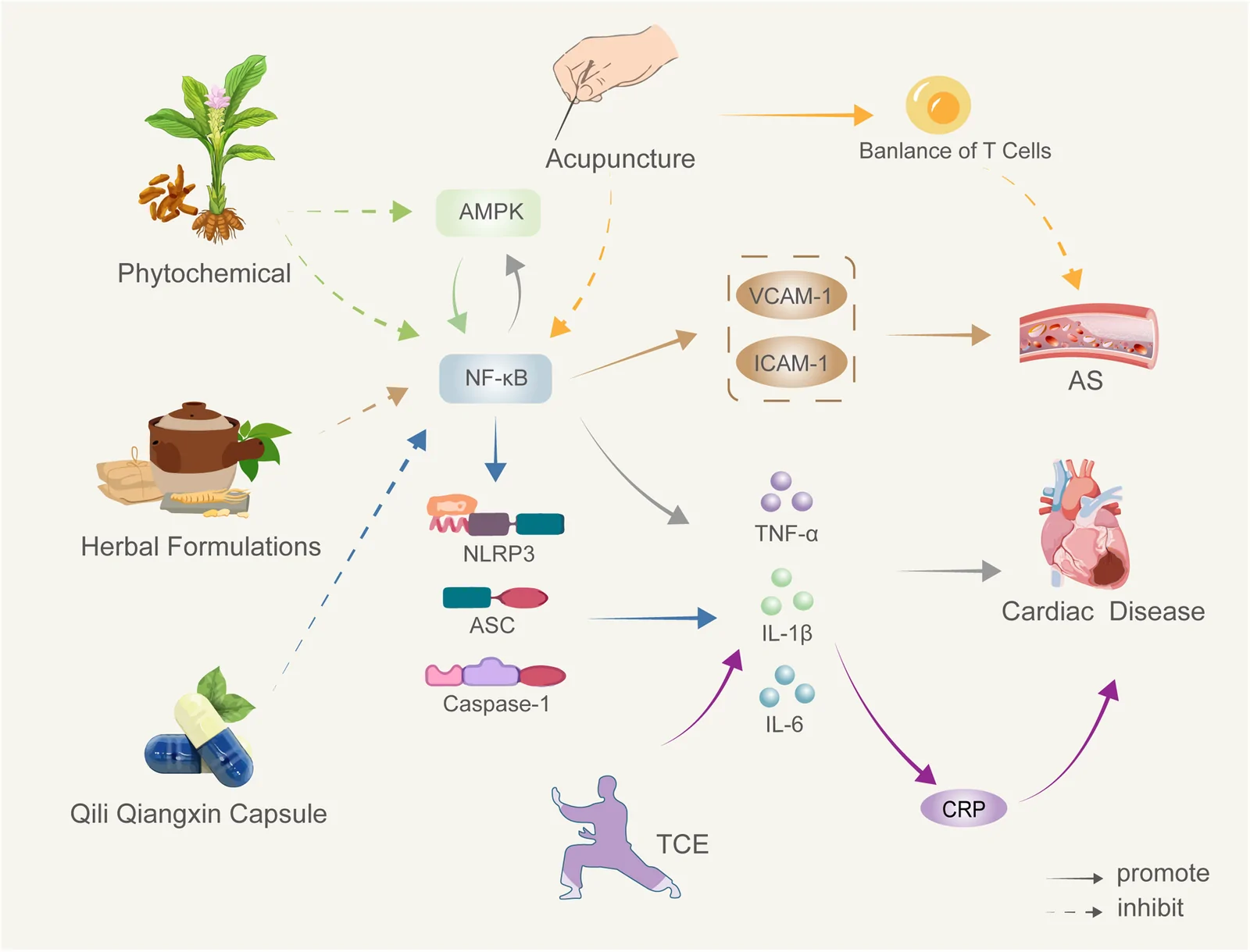
Core mechanisms of TCM in CR. CVD, cardiovascular diseases; GSH-Px, glutathione peroxidase; HO-1, heme oxygenase-1; NOX2, NADPH oxidases 2; NOX4, NADPH oxidases 4; NQO1, NAD**(P)** H quinone dehydrogenase 1; RAAS, renin-angiotensin-aldosterone system; ROS, reactive oxygen species; SCFAs, short-chain fatty acids; SNS, sympathetic nervous system; SOD, superoxide dismutase; T2DM, type 2 diabetes mellitus; TCM, traditional Chinese medicine; TMA, trimethylamine; TMAO, trimethylamine N-oxide.

The mechanism underlying the role of TCM in CR entails modulation of interconnected pathophysiological networks rather than isolated signaling pathways. As illustrated in Figure 1, TCM comprehensively ameliorates the core pathogenesis of CVD. This is achieved by reestablishing systemic homeostasis: dampening chronic inflammation, oxidative stress, mitochondrial dysfunction, and modulating the gut microbiota. Concurrently, TCM exerts direct cardioprotective effects by optimizing cardiomyocyte energy metabolism and restoring autonomic nervous system homeostasis while mitigating psychosocial stress that exacerbates cardiac burden. Via these synergistic, multi-targeted mechanisms, TCM ultimately improves long-term CR outcomes in patients with CVD.

### 3.1 Modulating chronic inflammation

Chronic low-grade inflammation exacerbates CVD progression primarily through the activation of the NF-*κ*B pathway and the NLRP3 inflammasome (27–29). TCM systematically targets these interconnected pathways to restore immune homeostasis.

At the molecular level, herbal formulations interfere with inflammatory signaling at multiple nodal points. Xuefu Zhuyu Decoction suppresses NF-κB p65 nuclear translocation, downregulating TNF-α, IL-1β, and endothelial adhesion molecules (VCAM-1, ICAM-1), thereby blocking monocyte infiltration and stabilizing atherosclerotic plaques (30). Similarly, Qili Qiangxin Capsule targets the NLRP3/ASC/Caspase-1 axis to attenuate IL-1β-mediated myocardial fibrosis in HF (31). Furthermore, bioactive ingredients (e.g., curcumin, paeoniflorin, notoginsenoside R1) can jointly suppress the NF-*κ*B/MAPK signaling pathway and upregulate IL-10 expression, ultimately delivering direct protective actions against cardiomyocyte injury (32–34).

**Figure 2:**
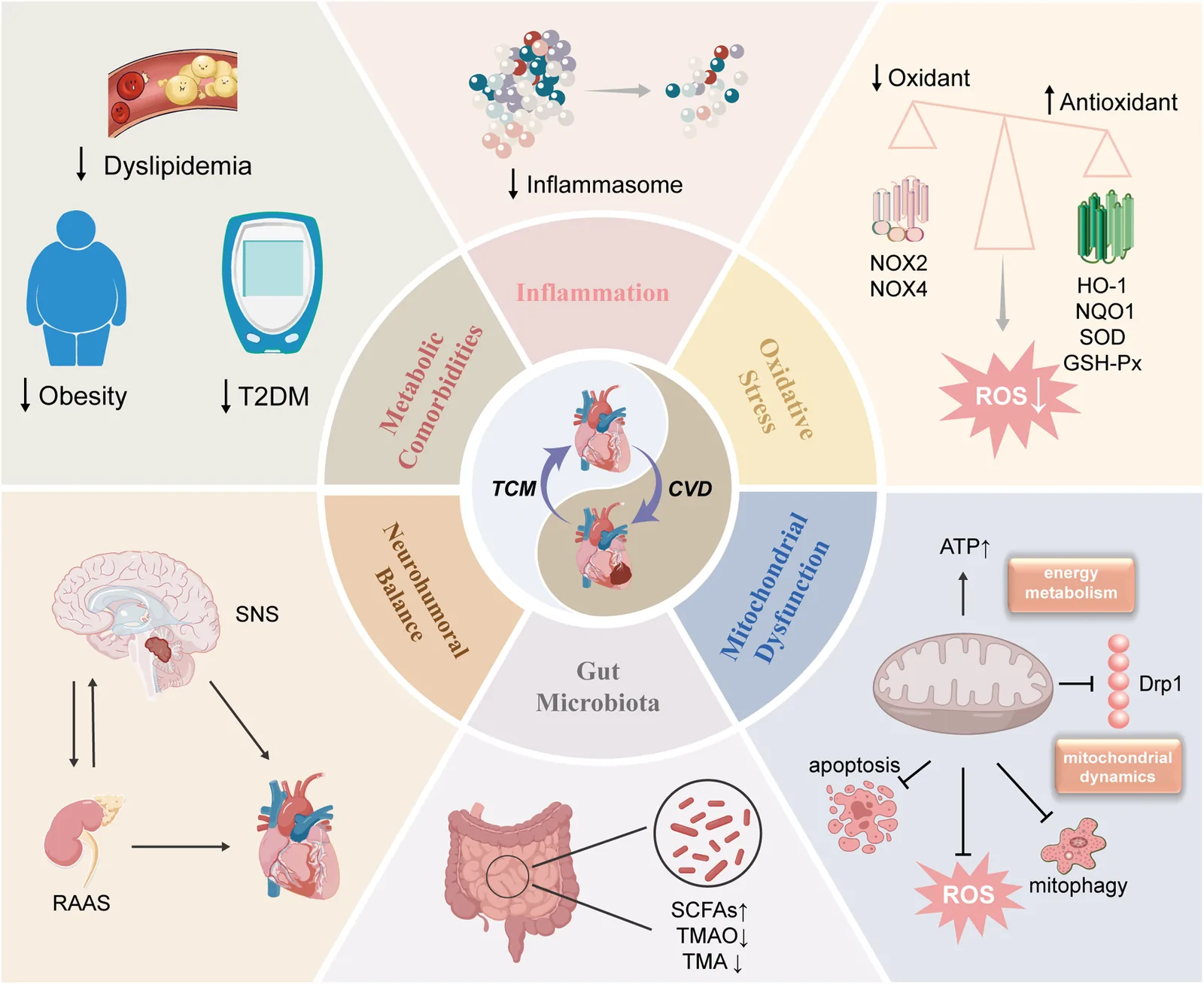
Anti-inflammatory effects of TCM in CR. AS, atherosclerosis; CRP, C-reactive protein; TCE, traditional Chinese exercises.

Beyond herbal medicine, acupuncture elicits anti-inflammatory responses primarily through somatosensory neuroimmune pathways. Specifically, acupoint stimulation at (e.g., PC6 and ST36) modulates T lymphocyte subsets and lower circulating TNF-α and IL-6, thereby alleviating plaque inflammation (35). In addition, TCE (including Tai Chi and Baduanjin) can downregulate CRP and IL-6 concentrations, providing an alternative non-pharmacological modality against myocardial inflammatory injury (36) (Figure 2).

### 3.2 Mitigating oxidative stress

Oxidative stress, triggered by excessive reactive oxygen species (ROS) and impaired antioxidant capacity, accelerates CVD progression by inducing lipid peroxidation, endothelial damage, and cardiomyocyte apoptosis (37). TCM counteracts this pathological cascade by systematically regulating core oxidative signaling axes.

At the molecular level, herbal formulations block ROS generation at the source. Buyang Huanwu Decoction suppresses NADPH oxidase (NOX2/NOX4) isoforms and reverses eNOS uncoupling, lowering ROS generation and restoring nitric oxide bioavailability to retard atherosclerosis progression (38). In addition, Danshen Yin activates the master regulatory Nrf2/ARE pathway, upregulating downstream antioxidant enzymes (HO-1, NQO1, SOD, GSH-Px) to clear excess ROS and attenuate myocardial oxidative injury (39). Furthermore, purified bioactive constituents (e.g., tanshinone IIA, puerarin, and astragalus polysaccharide) simultaneously suppress NOX activity and stimulate Nrf2 activation. This dual-targeted intervention interrupts the vicious cycle of ROS-induced ROS release, maintains mitochondrial structural integrity and inhibits cardiomyocyte apoptosis (40–42).

**Figure 3:**
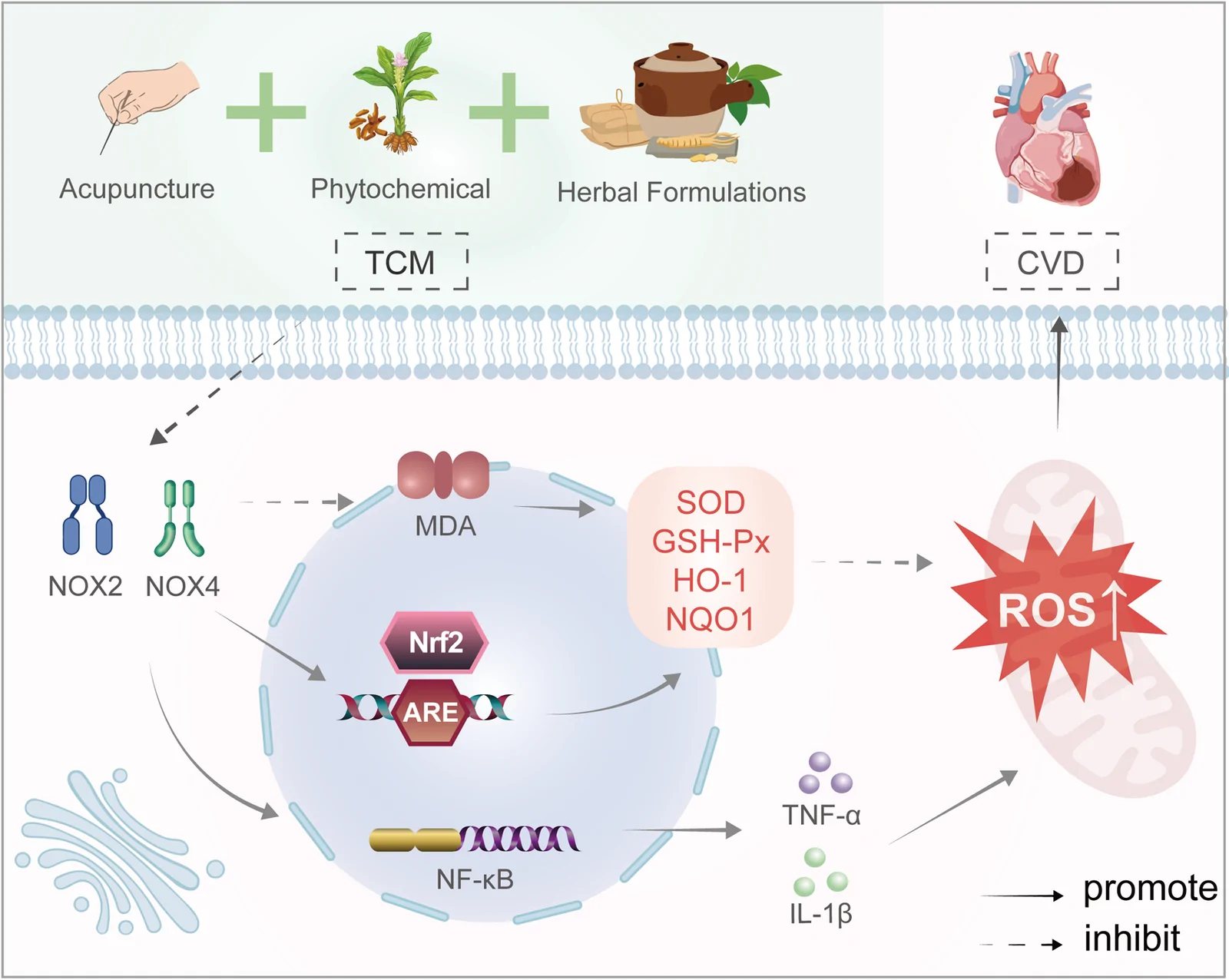
Anti-oxidative stress effect of TCM in CR. CVD, cardiovascular diseases; GSH-Px, glutathione peroxidase; HO-1, heme oxygenase-1; IL-1β, interleukin-1β; MDA, malondialdehyde; NF-κB, nuclear factor-kappa B; NOX2, NADPH oxidases 2; NOX4, NADPH oxidases 4; NQO1, NAD(P) H quinone dehydrogenase 1; ROS, reactive oxygen species; SOD, superoxide dismutase; TCM, traditional Chinese medicine; TNF-α, tumor necrosis factor-alpha.

Beyond pharmacotherapy, somatic interventions strengthen endogenous antioxidant defense systems. Acupuncture at acupoints (e.g., PC6, CV17, BL15) markedly increases myocardial SOD and GSH-Px activity and inhibits excessive MDA and ROS production under ischemia-reperfusion conditions. By inducing HO-1 expression and reducing oxidative-inflammatory mediators, acupuncture robustly alleviates oxidative myocardial damage and facilitates the recovery of cardiac function (43) (Figure 3).

### 3.3 Improving mitochondrial function

Acting as more than a metabolic “powerhouse” for energy generation, mitochondria are pivotal regulators of calcium dynamics, immunity, and programmed cell death. Mitochondrial dysfunction is particularly harmful to energy-consuming organs such as the heart, playing a pivotal role in the pathogenesis of CVD. Therefore, the development of specific therapeutic strategies targeting mitochondrial dysfunction in cardiomyocytes has garnered widespread attention (44). Notably, previous studies have demonstrated that TCM alleviates mitochondrial dysfunction via multi-pathway and multi-target mechanisms, thus mitigating cardiomyocyte injury and conferring cardioprotective effects (45). Its core regulatory actions involve modulating energy metabolism, clearing excess ROS, tuning mitophagy, normalizing mitochondrial dynamics, and suppressing apoptosis — effects achievable through diverse TCM modalities, including compound herbal prescriptions, single medicinal herbs, purified monomers, and non-pharmacological TCM interventions.

Compound Chinese herbal prescriptions are extensively applied for the prevention and clinical management of CVD, owing to their potential to alleviate impaired mitochondrial function. In detail, Qili Qiangxin Capsules exert protective effects on mitochondrial dynamics and mitophagy via restraining Drp1 activity and tuning the AMPK/mTOR signaling cascade (46). Likewise, Shengmai Powder restrains excessive mitochondrial fission by interfering with the Ca^2+^/Drp1 axis (47). Meanwhile, Shexiang Baoxin Pills eliminate excess ROS and repress mitochondrial intrinsic apoptotic signaling via activation of the PI3 K/AKT pathway (48). Collectively, accumulating studies demonstrate that these herbal formulations attenuate myocardial damage and relieve clinical symptoms linked to HF and myocardial ischemia-reperfusion injury.

Single Chinese herbs and their bioactive monomers have emerged as popular research topics owing to their identifiable chemical constituents and well-defined molecular targets, as they restore impaired mitochondrial function via multiple signaling pathways. In particular, ginsenoside Rb3 triggers activation of the PPAR–RXRα cascade to rebalance mitochondrial energy turnover (49). Astragaloside IV, on the other hand, stabilizes the mitochondrial membrane potential and reduces mitochondrial structural damage (50). In parallel, berberine rescues mitochondrial homeostasis through activation of the AMPK/PGC-1α signaling cascade (51). In addition, tanshinone IIA modulates mitophagy and eliminates excess ROS to attenuate mitochondrial oxidative injury (48). Ultimately, ginsenoside Rg1 safeguards mitochondrial homeostasis via triggering FUNDC1-dependent mitophagic signaling, which accelerates the removal of dysfunctional mitochondrial fragments (52).

TCM non-pharmacological interventions can also indirectly restore mitochondrial function to confer systemic cardioprotection. Accumulating evidence demonstrates that stimulation of specific acupoints including Neiguan (PC6), Xinshu (BL15) and Tanzhong (CV17) modulates the PI3K/AKT and AMPK signaling cascades, normalizing mitochondrial energy metabolism and suppressing mitochondrial apoptotic signaling (53). Studies have demonstrated that Tai Chi and Baduanjin facilitate mitochondrial biogenesis, boost mitochondrial energy metabolic efficiency, and suppress ROS overproduction to attenuate oxidative myocardial injury (54).

### 3.4 Regulating the gut microbiota

A growing number of animal and human studies have demonstrated that the composition and metabolic profiles of the gut microbiota are implicated in the pathogenesis of CVD, particularly hypertension, atherosclerosis, and HF (55). Of note, investigations targeting gut microbiota represent a novel frontier for decoding the underlying mechanisms of TCM, which bridges the regulatory pathways of TCM interventions with the clinical management of CVD (56).

Leng and colleagues demonstrated that Bushen Huoxue Decoction protects intestinal epithelial barrier homeostasis, which alleviates pathological cardiomyocyte hypertrophy and recovers overall cardiac performance (57). Furthermore, Suxiao Jiuxin Pills, a classic oral Chinese patent medicine widely applied for cardiac disorders, confer favorable cardiovascular protective benefits. In rat models of isoproterenol-triggered acute MI, this formulation recovers the depleted richness and diversity of intestinal microbial communities, revealing that its anti-cardiovascular injury activity relies partially on modulating gut microecological (58). Likewise, accumulating evidence confirms that Fuzi Decoction reshapes the synthesis of short-chain fatty acids in preclinical chronic heart failure (CHF) models. These microbial metabolites are universally recognized as protective mediators maintaining host cardiovascular homeostasis (59).

In parallel, growing preclinical data confirm that isolated bioactive herbal monomers and crude extracts can remodel gut microecological balance. As an example, pelargonidin, a plant-derived polyphenol, decreases systemic trimethylamine N-oxide levels and suppresses inflammatory infiltration within atherosclerotic plaques in ApoE^−^/^−^ mice (59, 60). In addition, berberine blocks choline-dependent generation of trimethylamine and trimethylamine N-oxide in both C57BL/6J wild-type and ApoE^−^/^−^ atherosclerotic mice, which alleviates pathological aortic plaque formation (61). In contrast, quercetin normalizes glucose metabolic dysfunction and insulin resistance through reconstructing balanced gut microbiota, thus lowering the risk of cardiovascular events (62).

Of note, accumulating studies have demonstrated that both electroacupuncture and manual acupuncture can reshape gut microbial community structure in spontaneously hypertensive rats (SHR). Such interventions elevate the relative abundance of beneficial genera (e.g., *Blautia*) and reduce that of pathogenic bacteria (e.g., *Helicobacter*), thereby markedly lowering systolic and diastolic blood pressure in SHR (63). Moreover, a randomized controlled trial reported that Baduanjin practice relieves clinical manifestations among elderly patients with stable angina pectoris by modulating gut microbiota and decreasing serum trimethylamine N-oxide (TMAO) concentrations (64). Collectively, these consistent findings underscore that TCM exerts crucial functions in CR through gut microecological regulation. Further rigorous research is required to fully decipher the molecular mechanisms by which Chinese herbal medicines treat CVD via remodeling gut microbiota.

### 3.5 Regulating neurohumoral balance

Neurohumoral overactivation, manifested as excessive activation of the renin–angiotensin–aldosterone system (RAAS) and sympathetic nervous system (SNS) coupled with parasympathetic withdrawal, acts as a vital upstream trigger for CVD progression and end-organ injury (65). TCM interventions can reverse such pathological aberrations and restore cardiovascular homeostasis.

From a pharmacological perspective, TCM prescriptions constructed under the “qi-tonifying and yang-warming” therapeutic framework exert robust regulatory effects on neurohumoral systems. As a representative agent, Qili Qiangxin Capsules blunt systemic RAAS overactivation by suppressing the synthesis of renin, Ang II and aldosterone, which abolishes Ang II-induced vasoconstriction and pathological cardiomyocyte hypertrophy. Importantly, this drug concurrently restrains excessive SNS signaling, limits catecholamine outflow, and cuts myocardial oxygen consumption, ultimately improving the prognosis of HF dual inhibition of RAAS and SNS axes has likewise been validated in classical formulas such as Tianma Gouteng Yin and Qingda Granules. Guided by the “liver-calming and wind-dampness extinguishing” therapeutic rule, these herbal recipes suppress Ang II receptor expression to lower blood pressure and slow progressive vascular and cardiac remodeling in hypertensive rodent models (66, 67).

Complementing its neurohumoral regulatory effects, TCM also directly acts on cardiac autonomic innervation. For example, acupuncture at acupoints PC6 and HT7 restores parasympathetic tone. By increasing heart rate variability and suppressing sympathetic overactivity, acupuncture raises the ventricular arrhythmia threshold and markedly improves cardiovascular prognosis (68). Likewise, Tai Chi efficiently restores autonomic equilibrium in patients with coronary heart disease (CHD), producing comparable increases in maximal oxygen uptake (VO_2_max) to standard moderate-intensity aerobic training (69).

### 3.6 Modulating systemic metabolism

Metabolic comorbidities, especially dyslipidemia and insulin resistance, act as major upstream triggers of atherosclerosis and pathological cardiac remodeling. Consistent with the holistic regulatory philosophy of TCM, various TCM interventions comprehensively counteract these metabolic risk factors and lower the incidence of recurrent cardiovascular events.

Pharmacological evidence reveals that multi-component TCM formulas restore disrupted lipid and glucose metabolism. Herbal mixtures containing bioactive constituents of *Crataegus pinnatifida*, *Cassia obtusifolia*, and *Alisma orientale* remodel circulating lipid profiles: they reduce harmful LDL-C and triglycerides and increase protective HDL-C, limiting lipid infiltration within vascular walls and stabilizing unstable atherosclerotic lesions (70). In parallel, bioactive compounds extracted from *Astragalus membranaceus*, *Coptis chinensis,* and *Pueraria lobata* activate insulin-dependent signaling to reverse insulin resistance and facilitate glucose translocation into target cells. Sustained glycemic homeostasis afforded by these herbal agents offsets chronic glucotoxicity, serving as a protective barrier against progressive cardiomyocyte and endothelial dysfunction (71).

Apart from medicinal treatments, TCM restores metabolic homeostasis by regulating daily lifestyle behaviors. TCE represented by Tai Chi and Baduanjin exert synergistic effects alongside tailored TCM dietary guidance (such as restricting excessive high-fat diet), which rectifies systemic energy homeostasis. Such multi-dimensional management decreases body mass index, relieves obesity-triggered hemodynamic overloading on the heart, and reconstructs aberrant metabolic profiles of patients (54). Clinical trial data further confirm that sustained Baduanjin practice achieves remarkable decreases in systolic and diastolic blood pressure, circulating triglycerides, and fasting glucose levels in hypertensive populations (72, 73).

Taken together, the cardioprotective mechanisms of TCM against CVD rely on multi-dimensional regulatory activities, covering inhibition of persistent inflammation, relief of oxidative injury, and maintenance of mitochondrial homeostasis. Meanwhile, gut microecological remodeling, normalization of neurohumoral status, and correction of abnormal systemic metabolic remodeling collectively construct the holistic regulatory network of TCM interventions for CVD. Crosstalk among these interlocking pathways exerts synergistic therapeutic benefits to halt pathological CVD progression, recover cardiovascular homeostatic balance, and optimize patient clinical outcomes. Such phenomena fully reflect the unique multi-targeted and holistic advantages of TCM in CR and cardiovascular disease intervention.

## 4 Application of TCM in CR

To convert the multi-target therapeutic merits of TCM into real-world clinical CR, systematic fusion of TCM modalities within CR frameworks is urgently required. This combined model supports individualized, dynamically modified rehabilitation schemes, which outperforms rigid unified rehabilitation programs in elevating long-term patient adherence.

### 4.1 TCE as a bridging rehabilitation modality

TCE are safe and effective interventions for CR. They have been widely applied in the rehabilitation of various CVD, including CHF, CHD, MI, and hypertension, providing diversified exercise modalities for patients with different exercise tolerance (Table 1).

**Table 1:** The application of TCE in CR.

Study ID and study design type	Sample size and intervention (Experimental/Control group)	Primary outcome measures and quantitative effect size (OR/RR/MD)	95% confidence interval (CI)	Adverse event rate
Coronary Heart Disease				
Li et al., 2022 Meta-Analysis (74)	1,025 cases (11 studies included) Baduanjin + Conventional CR vs. Conventional CR	MD = 2.83 (LVEF)	2.05–3.61 (LVEF)	Not reported
		MD = 6.67 (SAQ)	4.09–9.26 (SAQ)	
		SMD = 0.73 (SF-36)	0.55–0.91 (SF-36)	
		MD = −6.64 (SAS)	−7.69–5.22 (SAS)	
		MD = −6.63 (SDS)	−7.60–5.66 (SDS)	
Salmoirago-Blotcher et al., 2017 (75) RCT	29 participants (13 in PLUS group, 16 in LITE group)	**3 months:** MD = 100.33 (PA)	**3 months:** 15.70–184.95 min/week (PA)	Not reported
	Tai Chi (PLUS group: 3 sessions/week for 12 weeks + 12-week maintenance; LITE group: 2 sessions/week for 12 weeks)	**6 months:** MD = 111.62 min/week (PA)	**6 months:** 26.17–197.07 min/week (PA)	
		significant between-group differences in weight change and quality of life		
		no significant differences in aerobic fitness		
Chronic Heart Failure				
Ren et al., 2017 (76) Meta-Analysis	656 patients (11 trials included)	WMD = 65.29 m (6 MWD)	32.55–98.04 m (6 MWD)	Not reported
	Tai Chi + Conventional CR vs. Conventional CR	WMD = −11.52 points (QQL)	−16.5–6.98 points (QOL)	
		WMD = 9.94% (LVEF)	6.95–12.93% (LVEF)	
		SMD = −1.08 pg/mL (BNP)	−1.91–0.26 pg/mL (BNP)	
		WMD = −2.52 bpm (HR)	−3.49–1.55 bpm (HR)	
Hui et al., 2022 (77) Meta-Analysis	1,236 participants (15 studies included)	MD = −8.51 (MLHFQ)	−10.32 to −6.70 (MLHFQ)	Not reported
	Tai Chi + Usual Care vs. Usual Care Alone	MD = 43.47 m (6 MWT)	33.38 to 54.10 m (6 MWT)	
		MD = 6.07% (LVEF)	3.44 to 8.70% (LVEF)	
		SMD = −1.12 (BNP/NT-proBNP)	−1.70 to −0.54 (BNP/NT-proBNP)	
		MD = −2.89 (HAMD)	−4.87 to −0.91 (HAMD)	
		MD = −2.25 (PSQI)	−3.88 to −0.61 (PSQI)	
		MD = −1.34 s (TUGT)	−2.50 to −0.19 s (TUGT)	
		RR = 0.47 (HF hospitalization)	0.25 to 0.88 (HF hospitalization)	
		MD = 1.38 (VO2peak, non-significant)	−1.51 to 4.28 (VO2peak)	
Yang et al., 2023 (78) Meta-Analysis	1 RCTs included (total sample size not specified)	MD = −8.25 (overall QOL)	−13.62 to −2.89 (overall QOL)	Not reported
	Baduanjin Exercise + Conventional CR vs. Conventional CR	MD = 118.49 m (exercise capacity)	52.57 to 184.41 m (exercise capacity)	
		MD = −2.83 (physical domain)	−3.76 to −1.90 (physical domain)	
		MD = −2.52 (emotional domain)	−3.67 to −1.37 (emotional domain)	
		MD = −2.61 (general QOL)	−5.17 to −0.06 (general QOL)	
Dai et al., 2023 (74) Meta-Analysis	1,665 patients (21 RCTs included)	MD = 2.14 (VO2max)	1.02–3.26 (VO2max)	Not reported
	TCE (including Baduanjin, Tai Chi, etc.) + Conventional Rehabilitation vs. Conventional Rehabilitation	MD = 1.61 (AT)	1.06–2.16 (AT)	
		MD = 2.60% (LVEF)	1.17–4.02% (LVEF)	
		MD = 38.55 m (6 MWD)	36.67–40.42 m (6 MWD)	
		MD = 5.52 (QOL)	3.17–7.88 (QOL)	
		MD = 0.78 (tiredness and fatigue)	0.03–1.53 (tiredness and fatigue)	
		MD = 0.44 (shortness of breath)	0.26–0.62 (shortness of breath)	
		MD = 0.44 (facial puffiness and limb swelling)	0.12–0.76 (facial puffiness and limb swelling)	
		MD = 0.68 (palpitations)	0.14–1.21 (palpitations)	
Hypertension				
Li et al., 2024 RCT (79)	342 participants (173 in Tai Chi group, 169 in aerobic exercise group); 1,189 patients screened	**12 months:** MD = −2.40 mmHg (SBP)	**12-month:** −4.39 to −0.41 mmHg (SBP)	Not reported
	Tai Chi vs. Aerobic Exercise	**6 months:** MD = −2.31 mmHg (SBP)	**6-month:** −3.94 to −0.67 mmHg (SBP)	
		**24 h ambulatory:** MD = −2.16 mmHg (SBP)	**24 h ambulatory:** −3.84 to −0.47 mmHg (SBP)	
		**Nighttime ambulatory:** MD = −4.08 mmHg (SBP)	**Nighttime ambulatory:** −6.59 to −1.57 mmHg (SBP)	
Shao et al., 2020 (72) Meta-Analysis	1,058 patients (14 eligible RCTs)	MD = −8.52 mmHg (SBP)	−10.65 to −6.40 mmHg (SBP)	Not reported
	Baduanjin + Routine Treatment/Health Education vs. Routine Treatment/Health Education Alone	MD = −4.65 mmHg (DBP)	−6.55 to −2.74 mmHg (DBP)	
Xiong et al., 2015 (73) Meta-Analysis	8 RCTs	**vs. No Intervention:** WMD=−13.00 mmHg (SBP)	**vs. No Intervention:** −21.24 to −4.77 mmHg (SBP)	Not reported
	Baduanjin vs. No Intervention	WMD=−6.13 mmHg (DBP)	−11.20 to −1.07 mmHg (DBP)	
	Baduanjin vs. Antihypertensive Drugs	significant improvements in BMI, blood glucose, TG, LDL-C, HDL-C, QOL	**Vs. Antihypertensive Drugs:**	
	Baduanjin + Antihypertensive Drugs vs. Antihypertensive Drugs Alone	**vs. Antihypertensive Drugs:**	−2.07 to 4.17 mmHg (SBP)	
		no significant difference in SBP (WMD=1.05 mmHg) or DBP (WMD=1.90 mmHg)	−1.22 to 5.02 mmHg (DBP)	
		**Baduanjin + Antihypertensive Drugs vs. Antihypertensive Drugs Alone:**	**Baduanjin + Antihypertensive Drugs vs. Antihypertensive Drugs Alone:**	
		WMD=−7.49 mmHg (SBP)	−11.39 to −3.59 mmHg (SBP)	
		WMD=−3.55 mmHg (DBP)	−5.25 to −1.85 mmHg (DBP)	
		significant reductions in blood glucose and TC		
Myocardial infarction				
Li et al., 2021 (80) RCT	57 patients total (29 in Tai Chi treatment group, 28 in control group)	6 MWD: 552.97 ± 41.05 m (treatment group) vs. 508.29 ± 31.22 m (control group), t = 4.613, *P* < 0.01		No statistically significant difference in adverse events between the two groups (*P* > 0.05)
	Tai Chi + Conventional Treatment vs. Conventional Treatment Alone	QOL score was significantly higher in the treatment group (*P* < 0.05)		
Zhang et al., 2023 (81) Meta-Analysis	1,890 patients (21 eligible studies included)	MD = −96.34 (NT-proBNP)	−140.69 to −51.98 (NT-proBNP)	Not reported
	TCE (including Tai Chi, Baduanjin, etc.) + Conventional CR vs. Conventional CR Alone	MD = 4.58% (LVEF)	3.28 to 5.88% (LVEF)	
		MD = −3.83 mm (LVDD)	−5.27 to −2.38 mm (LVDD)	
		MD = −2.17 mm (LVESD)	−4.10 to −0.24 mm (LVESD)	
		MD = 69.60 m (6 MWT)	34.59 to 104.60 m (6 MWT)	
		MD = 4.38 (VO2)	2.25 to 6.51 (VO2)	
		MD = 13.34 (SF−36)	9.25 to 17.42 (SF−36)	
		MD = −4.34 (HAMA)	−5.18 to −3.50 (HAMA)	
		MD = −3.48 (HAMD)	−5.35 to −1.61 (HAMD)	
		RR = 0.31 (MACEs incidence)	0.20 to 0.47 (MACEs incidence)	

6MWD, 6-minute walking distance; 6MWT, 6-ninute walking test; BNP, B-type natriuretic peptide; DBP, diastolic blood pressure; GLU, glucose; HAMA, hamilton anxiety scale; HAMD, hamilton depression scale; HDL-C, high-density lipoprotein cholesterol; HR, heart rate; LVEF, left ventricular ejection fraction; LVDD, left ventricular end diastolic dimension; LVESD, left ventricular end systolic dimension; MACEs, major adverse cardiac events; MLHFQ, minnesota living with heart failure questionnaire; PA, physical activity; QOL, quality of life; RCT, randomized controlled trial; SBP, systolic blood pressure; SAS, self-rating anxiety scale; SAQ, seattle angina questionnaire; SDS, self-rating depression scale; SF-36, 36-item short form survey; TC, total cholesterol; TCE, traditional Chinese exercise; TG, triglyceride; VO_2_, oxygen consumption.

As a representative low-to-moderate-intensity TCE, Tai Chi presents multifaceted advantages in CR. Accumulating evidence verifies that Tai Chi integrated into CR regimens, either as monotherapy or adjunct to endurance training, produces synergistic cardioprotective effects and ameliorates long-term clinical outcomes among individuals with CVD (69, 74, 76). Primarily, Tai Chi improves cardiac functional performance in CHF cohorts, evidenced by elevated left ventricular ejection fraction, extended 6-minute walking distance, and decreased serum B-type natriuretic peptide (BNP) levels that alleviate cardiac load (76). Furthermore, this exercise normalizes autonomic equilibrium, augments aerobic capacity, and recovers arterial elasticity in CHD sufferers, with its VO₂max-improving efficacy comparable to routine low-to-moderate aerobic workouts (69). Economically accessible and linked to superior long-term adherence, Tai Chi exerts dual physical and psychological regulation via its unique mind-body harmony mechanism. It alleviates emotional disorders including anxiety and depression, filling the gap of insufficient psychological intervention in routine rehabilitation schemes, rendering it an ideal option for seniors and patients intolerant to strenuous exercise (74).

Baduanjin is a TCE comprising eight movements, each guided by unique TCM theories and exerting distinct physiological regulatory effects. A randomized trial conducted by Chen et al. delivers robust evidence supporting the application value of Baduanjin within CR. The outcomes revealed that regular Baduanjin training markedly alleviated fatigue and boosted quality of life among patients with CHF, offering a novel supplementary intervention for individuals living with chronic cardiovascular diseases (82).

In addition to Tai Chi and Baduanjin, other TCE including Yijinjing, Wuqinxi, and Liuzijue exert favorable therapeutic effects in CR. Clinical trials have confirmed that Yijinjing boosts cardiopulmonary fitness and stroke volume while lowering cardiovascular risk factors among patients with CHD (83). Wuqinxi decreases the incidence of MACEs in post-PCI patients and delivers supplementary benefits throughout CR. Meanwhile, accumulating evidence indicates that Liuzijue effectively alleviates hypertension (84).

In conclusion, TCE represented by Tai Chi, Baduanjin, Yijinjing, Wuqinxi, and Liuzijue deliver outstanding therapeutic benefits for CR, attributed to their low-to-moderate intensity and inherent mind-body harmony properties. In contrast, standard aerobic rehabilitation frequently suffers from unsatisfactory long-term adherence, especially for elderly vulnerable populations and patients complicated with severe left ventricular dysfunction. Under this circumstance, low- and moderate-intensity TCE constitute optimal transitional rehabilitation tools, permitting physically deconditioned cardiac patients to receive early, safe rehabilitation before engaging in rigorous standardized aerobic training programs.

### 4.2 Application of CHM in CR

CHM constitutes an indispensable branch of TCM and occupies a pivotal position in CR. Compound herbal formulas and single-herb extracts exert therapeutic actions through multi-targeted and multi-pathway mechanisms, which ameliorate cardiac function, relieve clinical symptoms, and elevate health-related quality of life.

#### 4.2.1 Chinese herbal compound prescriptions

Accumulating clinical and preclinical evidence regarding compound herbal formulas in CR validates their potent cardioprotective properties. As a typical proprietary herbal agent following the qi-tonifying and blood-circulating therapeutic principle, Yangxinshi Tablets exert cardiac protective impacts via orchestrating a spectrum of metabolic processes: glucose uptake, fatty acid turnover, and amino acid metabolic homeostasis (85). Cumulative clinical data demonstrate that adjuvant treatment with Yangxinshi Tablets combined with exercise rehabilitation remarkably elevates exercise capacity in post-PCI individuals, with objective quantification via cardiopulmonary exercise testing. Notably, this herbal preparation delivers prominent anxiolytic and antidepressant benefits for this patient cohort (11). Meanwhile, Shengmai San has been validated to generate robust cardioprotective outcomes for CHD patients manifesting qi-yin deficiency syndrome, through augmenting myocardial blood perfusion, reinforcing myocardial contractile performance, and restoring impaired cardiac function trial data further indicate that this herbal recipe relieves typical heart failure symptoms, optimizes overall cardiac function, and promotes disease-specific quality of life for HF sufferers (86). In parallel, preclinical research by Li et al. reported that Zhigancao Decoction attenuates pathological cardiac remodeling in experimental arrhythmia models (87). Separate clinical evidence shows that Xuefu Zhuyu Decoction effectively relieves symptomatic complaints, rectifies abnormal ECG indicators, and decreases recurrent angina episodes among CHD individuals (88).

#### 4.2.2 Single Chinese herb extracts

Great advances have been witnessed in translational research on single-herb extracts for CR. Astragaloside IV, a core bioactive compound of *Astragalus membranaceus*, has repeatedly been confirmed to reinforce antioxidative defense in cardiomyocytes, block apoptotic cell death, and deliver promising therapeutic efficacy for HF cohorts (12). As a key bioactive metabolite of Salvia miltiorrhiza, Danshensu repairs impaired endothelial function, dampens systemic inflammatory reactions, and prevents excessive platelet aggregation among CHD patients (13). In parallel, ginsenosides extracted from *Panax* ginseng augment myocardial systolic performance, strengthen resistance to hypoxic injury, and sustain stable cardiac performance, which collectively generates pleiotropic cardioprotective effects in populations with CHD and HF (14).

Distinct from standardized CR, TCM adopts syndrome differentiation and treatment as its core principle to target intertwined metabolic and mental comorbidities poorly controlled by conventional drug regimens. Stratified rehabilitation guided by TCM syndrome typing achieves individualized precise intervention, which amplifies therapeutic gains and reduces the incidence of adverse reactions among cardiovascular patients with multiple concurrent disorders.

### 4.3 External TCM therapies for early-stage rehabilitation

External TCM therapies constitute an important component of classic TCM interventions, including acupuncture, moxibustion, acupoint application, auricular acupressure, and herbal fumigation and washing. These approaches regulate the flow of qi and blood in the human body by stimulating specific acupoints or regions, thereby achieving therapeutic effects. In CR, physical exercise is frequently limited by hemodynamic instability and postoperative pain during inpatient (Phase I) and early outpatient (Phase II) rehabilitation. External TCM therapies demonstrate unique advantages during these phases and promising application prospects.

As safe non-pharmacological adjuncts, acupuncture and acupoint stimulation modulate autonomic function by targeting neuromodulatory acupoints such as PC6 and HT7, relieving postoperative angina and myocardial ischemia without increasing cardiac burden (43). Acupuncture at Neiguan (PC6) and Ximen (PC4) effectively reduces serum hs-cTnT levels and modifies heart rate variability indices, thereby improving autonomic nervous system activity in elderly patients with CHD after PCI (89). Meanwhile, this therapy also shows potential in alleviating clinical symptoms and relieving anxiety and depression in patients with stable angina pectoris (90). Combined with routine medications, acupuncture accelerates the transition to active rehabilitation.

A substantial body of evidence indicates moxibustion delivers warm stimulation via burning moxa sticks to unblock meridians and promote the circulation of qi and blood. Combined with heat-sensitive moxibustion, it can better improve cardiopulmonary exercise function and reduce NT-proBNP levels in patients with CHF (91). Furthermore, CR combined with moxibustion also significantly improves the overall effective rate of rehabilitation in patients with HF with favorable safety, making it suitable for the home rehabilitation stage (Phase Ⅲ) (91).

Taken together, embedding diverse TCM interventions within CR reconstructs rigid single-dimensional rehabilitation frameworks into an integrated biopsychosocial management system. Complementing the defects of rigid uniform rehabilitation protocols, combined TCM-CR models deliver all-round therapeutic advantages and serve as a promising clinical approach to facilitate long-term cardiovascular rehabilitation.

## 5 Challenges and prospects

Despite robust preclinical evidence, the widespread translation of TCM into CR faces distinct challenges. TCM focuses on holistic syndrome differentiation and treatment, while modern medicine concentrates on local pathological lesions and microscopic molecular changes, making it difficult to unify the evaluation criteria for outcome. Methodologically, evidence for TCM application in CR is commonly compromised by small sample sizes, inadequate randomization, and insufficient use of hard cardiovascular endpoints, such as MACEs and long-term mortality, thereby restricting its incorporation into international clinical guidelines. In addition, clinical implementation faces severe limitations. Dual-competency experts who master cardiology theory and practical TCM syndrome identification are in short supply, alongside a profound lack of standardized, replicable clinical protocols.

To overcome these barriers, future research must pivot toward methodological rigor. There is an urgent need for large-scale, multi-center, pragmatic randomized controlled trials that utilize standardized TCM interventions while adhering to established CR reporting standards. Furthermore, integrating multi-omics technologies (e.g., metabolomics, gut microbiome profiling) holds promise in transforming the TCM syndrome into a quantifiable biological phenotype, thereby bridging the gap between TCM and conventional precision CR. Ultimately, by establishing standardized clinical pathways, the integration of TCM and CR can evolve from regional empirical practice to a globally applicable rehabilitation strategy.
